# Rate of secondary HLH and performance of H-score in patients with severe COVID-19

**DOI:** 10.5339/qmj.2022.fqac.11

**Published:** 2022-04-04

**Authors:** Fiaz Alam, Karima Becetti, Laith Alamlih, Priyanka Cackamvalli, Safna Veettil, Basem Awadh, Mohamed Ibrahim, Samar Al Emadi

**Affiliations:** ^1^Division of Rheumatology, Department of Medicine, Hamad Medical Corporation, Doha, Qatar E-mail: FAlam1@hamad.qa; ^2^Qatar University, Doha, Qatar

**Keywords:** COVID-19, HLH, H-score

## Abstract

Background: Severe COVID-19 is thought to be caused by immune overdrive and cytokine storm. One of the cytokine storm syndromes frequently induced by infections is secondary hemophagocytic lymphohistiocytosis (HLH) which can be assessed using H-score. In this study, we aimed to evaluate the rate of patients with COVID-19 who meet HLH criteria based on H-score and the association of H-score with poor outcomes.

Methods: In a prospective cohort study of 19 patients with COVID-19 requiring ICU stay from March to May, 2020, we collected demographic and clinical data that focused on H-score's variables and COVID-19 outcomes. H-score ≥ 169 was used to determine the percentage of patients who met the HLH criteria. Mann-Whitney, Kruskal-Wallis, and Spearman rho tests and multiple regression analyses were carried out to evaluate the associated factors. The optimal H-score cut-off to predict poor COVID-19 outcome (need for intubation ± ECMO) was determined using receiver operating characteristic (ROC) analysis.

Results: In 669 patients with severe COVID-19 with a mean ± SD age of 50.3* ± *12.8 years, which comprised 95% men; 66% required intubation, 4% ECMO, and 16% died. Only 2% had an H-score ≥ 169. Patients with poor outcomes had a higher mean (SD) H-score than those without; intubation (96.0 [50.0] vs 75.0 [35.0], p < 0.01), ECMO (113.0 [25.0] vs 93.0 [50.0], p < 0.01) and death (98.0 [62.0] vs 93.0 [48.0], p < 0.01). Factors associated with H-score were diabetes (β coeff = − 10.4, p < 0.01), abdominal pain (β coeff = 19.1, p < 0.01), duration of COVID-19 symptoms (β coeff = − 0.7, p = 0.049), and days before ICU admission (β coeff = − 1.2, p = 0.01). H-score showed a fair ability to discriminate COVID-19 outcomes (AUC 0.61, 95% CI 0.54–0.67). An H-score of 85 was the optimal cut-off with a sensitivity 69% and 1-specificity 53%.

Conclusion: Despite its association with severity in COVID-19, H-score's ability to predict poor outcomes was only fair, indicating differences in the cytokine storm faced in COVID-19 compared with that during secondary HLH.

